# The reliability of repeated TMS measures in older adults and in patients with subacute and chronic stroke

**DOI:** 10.3389/fncel.2015.00335

**Published:** 2015-09-01

**Authors:** Heidi M. Schambra, R. Todd Ogden, Isis E. Martínez-Hernández, Xuejing Lin, Y. Brenda Chang, Asif Rahman, Dylan J. Edwards, John W. Krakauer

**Affiliations:** ^1^Motor Performance Laboratory, Department of Rehabilitation and Regenerative Medicine, Columbia UniversityNew York, NY, USA; ^2^Department of Biostatistics, Columbia UniversityNew York, NY, USA; ^3^Neural Engineering Group, Department of Biomedical Engineering, City College of New YorkNY, USA; ^4^Non-Invasive Brain Stimulation and Human Motor Control Laboratory, Burke-Cornell Medical Research InstituteWhite Plains, NY, USA; ^5^Brain, Learning, Animation, and Movement Lab, Department of Neurology, Johns Hopkins UniversityBaltimore, MD, USA

**Keywords:** TMS, reliability, standard error of the measurement, measurement error, smallest detectable change, ICC, biomarker

## Abstract

The reliability of transcranial magnetic stimulation (TMS) measures in healthy older adults and stroke patients has been insufficiently characterized. We determined whether common TMS measures could reliably evaluate change in individuals and in groups using the smallest detectable change (SDC), or could tell subjects apart using the intraclass correlation coefficient (ICC). We used a single-rater test-retest design in older healthy, subacute stroke, and chronic stroke subjects. At twice daily sessions on two consecutive days, we recorded resting motor threshold, test stimulus intensity, recruitment curves, short-interval intracortical inhibition, and facilitation, and long-interval intracortical inhibition. Using variances estimated from a random effects model, we calculated the SDC and ICC for each TMS measure. For all TMS measures in all groups, SDCs for single subjects were large; only with modest group sizes did the SDCs become low. Thus, while these TMS measures cannot be reliably used as a biomarker to detect individual change, they can reliably detect change exceeding measurement noise in moderate-sized groups. For several of the TMS measures, ICCs were universally high, suggesting that they can reliably discriminate between subjects. TMS measures should be used based on their reliability in particular contexts. More work establishing their validity, responsiveness, and clinical relevance is still needed.

## Introduction

Over the past three decades, increasing numbers of studies have used transcranial magnetic stimulation (TMS) to examine neurophysiology in pathology and health. Single- and paired-pulse TMS techniques probe the motor cortex and its connections, but do not themselves alter overall brain excitability (Kujirai et al., 1993; Nakamura et al., 1997; Chen, 2000, 2004; Chen and Udupa, 2009). It is generally assumed that change in the average motor evoked potential (MEP) amplitude, resulting from the same stimulation intensities, reflects true biological change in the corticospinal tract (CST), and/or intracortical circuitry (Rothwell, 2010). Although TMS has great potential for establishing physiological biomarkers, a rigorous appraisal of the quality of TMS as a measurement instrument has never been sufficiently undertaken. The instrument of TMS encapsulates the stimulation and recording setup and the operator. As a field, we have not established the degree to which TMS measurements are precise, accurate, or clinically relevant. Without this foundational knowledge, we risk ascribing meaningful neurophysiological mechanisms to meaningless TMS changes.

There have been hundreds of studies since the early 1990s using TMS to investigate neurophysiological change in healthy adults and stroke patients. To our knowledge, none placed their results in the context of the measurement error associated with TMS, instead judging results on the statistical assessment of differences associated with an intervention or time. The pervasive use of TMS, without questioning the quality of the measurements it produces, has perhaps led to a false sense of security that TMS is a superior measurement instrument. Work to prove this point, however, is largely lacking.

How do we judge if TMS is a good instrument for assessing neurophysiology? A useful measurement instrument is reliable and valid, producing data that are accurate and meaningful (Portney and Watkins, 2009). To gauge an instrument's utility, we must know how it fares in three main *domains* of instrument quality: reliability, validity, and responsiveness (Mokkink et al., 2010). In other words, TMS measures should produce stable measurements in unchanging subjects, the measures should tell us something about neurophysiology, and the measures should be able to detect change in neurophysiology. These instrument qualities should furthermore be characterized for different subject populations. In this study, we focused on characterizing the first quality domain of TMS—reliability—in older healthy subjects and stroke patients.

The lack of a comprehensive instrument assessment is not unique to TMS. Across health outcomes research, a major barrier to evaluating an instrument's utility has been disagreement about the terminology, definitions, and computations associated with each quality domain (Mokkink et al., 2010). For example, the term “reliability” has been used interchangeably with variability, consistency, reproducibility, precision, repeatability, agreement, and stability; depending on context and application, some of these terms represent distinct concepts operationalized by different mathematical formulae (Mokkink et al., 2010). The lack of a coherent vocabulary and taxonomy has led to a considerable misunderstanding of concepts, misuse of calculations, and misapplication of results for judging instrument quality, with TMS being no exception. In light of the confusion, we adopt the language developed by experts in clinimetrics, the methodological discipline focusing on the quality and use of measures in clinical medicine (de Vet et al., 2003; Mokkink et al., 2010).

Reliability is the degree to which repeated measures in unchanging individuals provide similar results (de Vet et al., 2006). A reliable instrument produces measurements that are consistent and error-free (Portney and Watkins, 2009; Mokkink et al., 2010). In stable individuals, reliability is the degree to which these measurements are the same over time (i.e., test-retest), as assessed by the same rater at different times (i.e., intra-rater), or as assessed by different raters at the same time (i.e., inter-rater) (Portney and Watkins, 2009; Mokkink et al., 2010).

For measurements taken at test-retest, reliability can address two different questions: how unchanging measurements are within individuals, or how unchanging individuals are relative to others. The domain of reliability is thus an umbrella term for distinct subtypes, so-called “measurement properties,” of reliability. The first measurement property of reliability is called measurement error, which assesses how good the agreement is between repeated measurements in an individual. It tells us how close measurements are with repeated testing in stable individuals (de Vet et al., 2006).

The second measurement property of reliability is called reliability_MP_, which assesses how well individuals can be distinguished from one another. It tells us how unchanging the positions of stable individuals are relative to each other at repeated testing (de Vet et al., 2006; Streiner and Norman, 2008). [N.B.: The duplicate use of the term “reliability” for both the *domain* of reliability and one of its *measurement properties* is undeniably confusing; despite debate, however, this duplication was upheld in light of historical usage (Mokkink et al., 2010). For clarity, we specify the *measurement property* of reliability with the subscript “MP.”].

When deciding which reliability measurement property to use for evaluating an instrument, the instrument's intended application needs to be considered (Guyatt et al., 1987; de Vet et al., 2006). If TMS is to be used for evaluation (e.g., “did Patient X's measurements change after an intervention?”), a small measurement error is needed (Guyatt et al., 1987; de Vet et al., 2006; Terwee et al., 2007). If TMS is instead to be used for diagnosis or staging (e.g., “is Patient X sicker than Patient Y?”), a high reliability_MP_ is needed (de Vet et al., 2006, 2011).

The majority of research with TMS uses it in an evaluative manner, for example, measuring neurophysiological change associated with time or an intervention. Knowing the measurement error associated with TMS is therefore critical for assessing its reliability. Knowing the smallest detectable change (SDC), derived from the measurement error, is its pragmatic extension. The SDC is the amount of change necessary to exceed measurement error (Beckerman et al., 2001). When the SDC is unknown, one cannot know if a change in measurements reflects a true change or simply measurement noise. An observed change less than the threshold value of SDC would be classified as measurement error.

Although generally not used in this manner, TMS also has diagnostic potential, for example, grading (i.e., staging) the severity of a lesion in the corticospinal tract. For this, reliability_MP_ should be known. Of note, most of the studies evaluating “reliability” in TMS have documented only reliability_MP_, despite TMS's primarily evaluative use.

We are not the first to undertake the important task of characterizing TMS reliability, but previous attempts in our view are questionable with respect to their methodologies and interpretations. For example, Pearson's correlation coefficient (r) has been used to identify the strength of association between test-retest values (Balslev et al., 2007). While correlation reflects the association between two measurements, it does not reveal the amount of sameness (i.e., agreement) between them. For instance, in a sample whose measurements are twice the magnitude at retest, correlation between measurements is perfect (i.e., *r* = 1), but there is no agreement. Others have used a Cronbach's alpha to document test-retest reliability (Farzan et al., 2010). Cronbach's alpha characterizes the consistency of multiple distinct measures probing the same construct, for example, how well the scores of the first half of questions correlate with the second half in a questionnaire (Mokkink et al., 2010; de Vet et al., 2011). Cronbach's alpha is not appropriate for assessing agreement between individual measurements from a single TMS measure over time.

Another common approach has been the use of inferential statistics to draw conclusions about measurement agreement. *T*-tests and ANOVAs have been used to test for statistical differences between repeated TMS measures in younger healthy subjects (McMillan et al., 1998; Boroojerdi et al., 2000; Maeda et al., 2002; Uy et al., 2002; Corneal et al., 2005), older healthy subjects (Wolf et al., 2004), and chronic stroke subjects (Liepert et al., 2000a; Butler et al., 2005). Based on non-significant differences between mean measurements, investigators have concluded that the measures are stable and reliable. Carl Sagan's warning is appropriate here: “the absence of evidence is not evidence of absence” (Sagan, 1995). Not detecting significant differences between measurements does not prove they are the same. Two or more distributions may show no statistical difference, but may be composed of pairs of measurements with no agreement. Thus, a non-significant difference between measurements does not imply high agreement or low measurement error.

A final problematic approach has been the widespread misunderstanding and misapplication of reliability_MP_. A high reliability_MP_ estimate is commonly misinterpreted as signifying low measurement error, with investigators deeming the TMS measure appropriate for evaluative use (Mortifee et al., 1994; Carroll et al., 2001; Kamen, 2004; Malcolm et al., 2006; Christie et al., 2007; Koski et al., 2007; Cacchio et al., 2009, 2011; Wheaton et al., 2009; Hoonhorst et al., 2014; Liu and Au-Yeung, 2014). This is simply wrong; the reliability measurement properties are not interchangeable and each implies a distinct concept. Additionally, reliability_MP_ is highly influenced by the dispersion of subjects in a sample. A sample with large between-subject variability will produce a high reliability_MP_ estimate for a measure, even despite a sizable within-subject measurement error. The influence of the sample's dispersion on a measure's reliability_MP_ thus constrains its generalizability: the reliability_MP_ estimate is appropriate for use only in samples with similar heterogeneity, a point that is rarely underscored.

In this study, we sought to establish the reliability of common TMS measures taken repeatedly from the FDI representation of healthy older adults and subacute and chronic stroke subjects. For TMS's evaluative and diagnostic applications, we estimated measurement error and reliability_MP_, respectively. We additionally derive the SDC for TMS measures, for practical use in future evaluative studies. We endeavored to make this manuscript operate as a primer for how one goes about assessing the qualities of a TMS measure, which requires detailed descriptions of TMS techniques and rigorous understanding of reliability assessments. It is our hope that with its transparency and detail, the manuscript can serve as a template for future reliability studies in TMS.

## Materials and methods

### General approach

Four identical testing sessions were used to assess test-retest reliability of MEP recordings from TMS applied to bilateral primary motor cortices. Subjects were studied on two consecutive days, in morning and afternoon sessions, 3.5–5 h apart. Sessions were performed at the same time each day within subject. Each session lasted 1.5–2 h. No experimental interventions were given between sessions.

Subject testing was conducted in the Motor Performance Laboratory at Columbia University and in the Non-Invasive Brain Stimulation and Human Motor Control Laboratory at Burke Rehabilitation Center. The study was approved by each facility's Institutional Review Board. The laboratories used identical hardware, software, equipment, and supplies in their neurophysiology setups, and differed only in the subject's chair and window view. A single operator (HS) conducted the assessments and data analysis. Each subject was tested at one site only.

### Subjects

Three separate groups of adults (total *n* = 62) were studied: healthy older adults (*n* = 21), subacute stroke subjects (*n* = 20), and chronic stroke subjects (*n* = 21). Healthy older adults were included because they often serve as stroke study controls, given the increased incidence of stroke with age (Sacco et al., 1997), and because their neurophysiology is different from that of younger adults (Rossini et al., 1992; Peinemann et al., 2001; McGinley et al., 2010). Subacute and chronic stroke groups were used because both recovery epochs have been probed with TMS (Bütefisch et al., 2003, 2008; Hummel et al., 2005; Hummel and Cohen, 2005; Liepert, 2006; Swayne et al., 2008; Khedr et al., 2009, 2013; Takechi et al., 2014). All subacute strokes, 5 chronic stroke, and 6 healthy older subjects were tested at Burke; all other chronic stroke and healthy older subjects were tested at Columbia.

Subjects were included if they were ≥40 years old, were able to give informed consent, and if stroke patients, had a single ischemic stroke resulting in paresis < 6 months (subacute stroke group) or ≥6 months previously (chronic stroke group). Because we wanted to obtain TMS outcomes in both hemispheres, only subjects who could at least marginally abduct their paretic index finger (MRC ≥ 1) and who had a recordable TMS-evoked response were included. Psychoactive medications (SSRI, SNRI, or bupropion) were permitted as long as they were taken consistently. Stroke subjects were excluded for preserved motor strength in the upper extremity (i.e., full strength on manual motor testing including no pronator drift, orbiting, or reduced finger individuation) or receptive aphasia; all subjects were excluded for any major medical, psychiatric, or non-stroke neurological condition that could interfere with motor function assessment or participation; history of seizure, neurosurgery, traumatic brain injury, or substance abuse; or thoracic or intracranial metal objects, implants, or devices, except for dental work. All subjects gave written informed consent to participate, in accordance with the Declaration of Helsinki.

### Psychophysical and clinical characteristics

Following each session, subjects reported levels of alertness during the session and excitement to participate on a scale from 1 to 10, with 10 as maximum. They also reported quantity of pre-testing sleep, exercise, and caffeine intake. Following session 3, subject demographics, clinical history, and handedness [Edinburgh Handedness Inventory; +1 and −1 indicate dominance for right and left hand, respectively (Oldfield, 1971)] were obtained. A neurological examination of the upper extremities, including the assessment of bilateral upper extremity strength by Medical Research Council (MRC) scale (Medical Research Council of U.K, 1978), was also performed at that time by a neurologist (HS). Stroke risk factors (hypertension, atrial fibrillation, coronary artery disease, diabetes, hypercholesterolemia, and tobacco use) and current medication use were documented.

### TMS measures

The two neurophysiology laboratories had an identical setup and equipment. Subjects were seated comfortably in an office chair with their forearms relaxed on a lap pillow. Arms were consistently positioned across sessions. Frameless stereotaxic equipment (Brainsight, Rogue Research, Canada), used to co-register the subject's scalp positions with a phantom MRI brain image, ensured stimulation accuracy during and across sessions. Co-registration errors to the phantom's surface landmarks were matched to ≤3 mm at each follow-up session.

Surface EMG was obtained from bilateral first dorsal interosseous (FDI) muscles, with electrodes taped in a belly-tendon orientation (SX230-100 and K800; Biometrics Ltd, UK). The integrated electrode contains two poles at a fixed distance of 2 cm. To ensure consistent electrode placement across sessions, electrodes were outlined with permanent ink on the skin and subjects were advised not to scrub the area. The EMG signal was sampled at 1000 Hz, amplified 1000x, band-pass filtered at 15–450 Hz, and saved for offline analysis. All assessments were taken at rest, and EMG activity was monitored online to ensure muscle relaxation.

TMS was delivered to the cortical hand representation of the motor cortex (M1), using Magstim BiStim^2^ and a 70-mm figure-of-eight remote control coil (Magstim Company Ltd, UK). Stimulation intensity determinations and data acquisition were performed in BiStim mode. Pulses were generated using specialized software (Signal; Cambridge Electronic Devices, UK) and a 1401 microprocessor (Cambridge Electronic Devices, UK). The TMS coil was held tangentially to the skull with the coil handle pointed 45° posterior-laterally to the sagittal plane, which orients the coil approximately perpendicular to the central sulcus and induces a posterior-to-anterior current direction (Pascual-Leone et al., 1994; Ruohonen and Ilmoniemi, 1999). A search in a ~1 cm-step grid pattern at ~50 percent of the maximum stimulator output (%MSO) was conducted to grossly identify the area producing the largest amplitude MEP (the “hotspot”) for the contralateral first dorsal interosseous (FDI) muscle. Resting motor threshold (rMT), the %MSO eliciting at least 5 out of 10 MEPs ≥50 μV, was obtained at that location. Using this %MSO and centering on this preliminary hotspot, a repeated grid search and new rMT determination refined the localization of the hotspot. The hotspot was virtually marked on the phantom brain and used at subsequent sessions, though its position was confirmed physiologically each time. If a superior hotspot was found at retest, this position was marked and followed. Almost always, the hotspot did not change; occasionally, the coil position required a minor adjustment in roll or pitch.

In healthy subjects, the left FDI hotspot was always probed first. In stroke subjects, the non-lesioned FDI hotspot was always probed first. The order of the TMS measures was purposefully fixed so that an order effect, if present, would have a consistent influence on measurement variability. If an order effect were present, for example from fatigue, then randomization would introduce additional measurement noise, i.e., by testing a subject who is alert for the measure at one session and fatigued at the next. Our approach was also most representative of a typical pre-post testing design, in which the order of outcome measures is not varied across sessions or individuals. We chose TMS measures based on their common usage in TMS studies, not necessarily based on their potential validity.

All TMS stimuli were delivered at an inter-trial interval of 7 s. Recruitment curves (RC) were generated from 10 stimuli each given at ascending stimulation intensities of 100, 110, 130, 150, and 170% rMT, or until 100% MSO was reached. MEP amplitudes were fitted to a Boltzmann sigmoid function (Carroll et al., 2001)

$$\begin{array}{l} y\left(x\right)=\frac{plateau}{1+e^{\frac{S_{50}-x}{slope}}} \end{array}$$

to estimate component RC parameters. Recruitment curve *plateau* is the maximum amplitude (mV), *S*_50_ is the stimulus intensity *x* (%MSO) required to evoke a response equal to half the maximum amplitude, and *slope* is the MEP amplitude increase with each percentage point of stimulator intensity increase (mV/%MSO). Model parameters ($\vec{\theta }=\left\{plateau,S_{50},slope\right\}$) were estimated with a standard least squares curve-fitting algorithm (trust-region-reflective algorithm) in MATLAB 8.1 (MathWorks Inc., USA). The parameter estimation method minimizes the root mean square error

$$\begin{array}{l} \vec{\theta }=\underset{\vec{\theta }}{\mathrm{argmin}}\sqrt{\frac{1}{N}\overset{N}{\underset{i=1}{\sum }}{\left[d_{i}-y\left(x_{i}|\theta \right)\right]}^{2}} \end{array}$$

between the actual MEP amplitudes (*d*) and the estimated amplitude *y*(*x*|θ) given a set of parameters. The amplitude of *plateau* was bounded to ≤8 mV to provide a physiologically plausible approximation of the maximum MEP amplitude. All other parameters were left unconstrained.

Short-interval intracortical inhibition (SICI) and intracortical facilitation (ICF) were generated with a conditioning stimulus (CS) delivered 2 or 10 ms prior to a test stimulus (TS), respectively (Kujirai et al., 1993). SICI and ICF were tested at CS intensities of 60 and 80% rMT to identify which CS intensity produced more reliable measurements; these measures are henceforth specified as SICI_60_ and ICF_60_ or SICI_80_ and ICF_80_. For TS, the stimulator intensity (TS_MSO_) was adjusted to produce an MEP (TS_MEP_) ~1 mV in amplitude. If this size could not be achieved, particularly in stroke subjects, the TS_MSO_ was set to the stimulation intensity above which no further increases in TS_MEP_ amplitude could be found (Swayne et al., 2008). Ten trials each of TS_MEP_, SICI, and ICF were recorded in repeating order for each CS intensity. Long-interval intracortical inhibition (LICI) was obtained with two stimuli separated by 100 ms (Nakamura et al., 1997). Both stimuli followed the same TS_MEP_ determination as above. Ten trials each of alternating TS_MEP_ and LICI were recorded. For SICI, ICF, and LICI, the average amplitude of conditioned MEPs were normalized to the average amplitude of unconditioned TS_MEP_ [i.e., (CS+TS)/TS], and are reported as a decimal fraction of TS_MEP_.

For reliability assessments of TS_MEP_, the MEP amplitudes of the 30 TS trials in each session (i.e., the three sets of 10 TS trials used to calculate SICI_60_/ICF_60_, SICI_80_/ICF_80_, and LICI separately) were averaged. For TS_MSO_, only the first %MSO was used, in order to exclude stimulator intensity adjustments that may have occurred within session.

Following TMS assessment in a hemisphere, pinch force was obtained from its contralateral hand. Subjects sat with shoulder adducted, elbow flexed at 90°, forearm midway between pronation and supination, and wrist in ~15° extension. Subjects held the force transducer (P200; Biometrics, UK) between the pad of thumb and radial side of the flexed index finger (i.e., a lateral or key pinch). Three maximal voluntary contractions (MVC) were held for 3 s each, with 10–20 s rest between, and stored offline. The maximum voltage of the force was extracted with a custom-made Signal script, and a conversion of 11.34 kg/V was applied. The trials were averaged within and then across sessions for each hand.

#### TMS measurement analysis

For TMS measurements, peak-to-peak MEP amplitude was measured using a custom-made script (Gray, 2015). Trials were discarded if EMG activity exceeded 100 μV in the 250 ms prior to TMS stimulus delivery. Three additional stimuli to be discarded were built into the beginning of each assessment, to eliminate the influence of excessively large MEPs commonly seen with initial stimuli and to allow time for coil positioning.

For RC parameter estimations, the curve fits to the raw MEP amplitudes for each session were visually examined to confirm the appropriate fit of the model to the data. The hemisphere's RC parameter estimations were discarded for any session where the curve appeared exponential or the plateau value equaled the upper parameter boundary (plateau = 8 mV).

### Reliability measurement properties

#### Variance decomposition

Classical test theory (CTT) postulates that an observed measurement is composed of the true measurement and an error term of the measurement (Lord and Novick, 1968). By CTT assumptions, the variance of the observed measurement is composed of the variance in the true measurement and the variance in the error term (de Vet et al., 2011):

$$\begin{array}{l} \sigma_{observed}^{2}=\sigma_{true}^{2}+\sigma_{error}^{2} \end{array}$$

Since the available data involve multiple observations on the same subjects, it is possible to decompose the variances of measurements from each hemisphere into several components and, by fitting statistical models that include random effects, to obtain estimates for the variance of each component. In particular, the “error” component can consist of between-subjects variability of measurements made on different subjects, day-to-day variability of measurements made on the same subject, replication variability of measurements made on the same subject within the same day, and “residual” variance, which captures all other sources of variability.

Based on our analysis of the data, we did not include the replication variance (measurements made on the same subject within the same day) in our final models. Because there are only two replications per day per subject, there are insufficient data to obtain reliable estimates of a time effect. Furthermore, estimates of this variance component were consistently very small. Therefore, we omitted the random effect of time in the model, effectively combining that variance with the residual variance.

For the purposes of calculating reliability measurement properties, a random effects model was used. Fixed effects, if any, should be considered a part of measurement error (de Vet et al., 2006). In most experimental contexts, testing days, and raters are assumed to be randomly chosen and representative of their class, and are expected to have similar fixed effects in future studies (de Vet et al., 2006, 2011). Excluding systematic differences could be appropriate if ranking subjects over time is intended (Terwee et al., 2007), but this has never been an application of TMS measures.

In our model, the variance of our measures was decomposed for each hemisphere as:

$$\begin{array}{l} \sigma_{observed}^{2}=\sigma_{subjects}^{2}+\sigma_{days}^{2}+\sigma_{residual}^{2} \end{array}$$

where $\sigma_{subjects}^{2}$ is the between-subject variance, $\sigma_{days}^{2}$ is the between-days variance, and $\sigma_{residual}^{2}$ is the error term that captures the remaining unexplained variability and includes interactions between all predictors (McGraw and Wong, 1996; de Vet et al., 2006).

#### Measurement error: standard error of the measurement (SEMeas) and relative SEMeas (SEMeas%)

The decomposed variances were used to estimate measurement error. Measurement error, also known as agreement (de Vet et al., 2006), is the spread of repeated measurements due to systematic and random error, not due to true changes in the construct being measured (Terwee et al., 2007; Mokkink et al., 2010). In other words, the measurement error estimates the “spread” or “noisiness” of repeated measures within stable individuals. The smaller the measurement error, the less variable and the more reliable the measure (Harvill, 1991; Tighe et al., 2010). The measurement error is particular to the measure and the population from which it is taken, not to the instrument universally. One would therefore expect the measurement errors of TMS measures to differ from one another, and for their measurement errors to change with pathology, age, and cortical muscle representation.

Measurement error is estimated by the standard error of the measurement (SEMeas; N.B.: it is generally abbreviated as SEM, but we chose to permute the acronym to avoid confusion with standard error of the mean.) SEMeas is the standard deviation of all within-subject sources of variance and excludes between-subject variance. It is calculated as:

$$\begin{array}{l} SEMeas=\sqrt{\sigma_{day}^{2}+\sigma_{residual}^{2}} \end{array}$$

The SEMeas has some unique attributes that make it particularly helpful in assessing instrument reliability. First, SEMeas is uninfluenced by the heterogeneity of sample from which it was derived, because between-subject variability is not included in its estimation (Tighe et al., 2010). Thus, it is considered a concrete property of the measure for the sampled population (Nunnally and Bernstein, 1994; Weir, 2005). Second, the SEMeas is largely stable across the spectrum of measurements for a measure (Harvill, 1991; Nunnally and Bernstein, 1994). Finally, the SEMeas is expressed in the same metrics as the measurement, providing ease of interpretation (Nunnally and Bernstein, 1994; Wyrwich et al., 1999).

The relative SEMeas (SEMeas%) is similar in concept to coefficient of variation in that, normalizing to the measurement mean, it provides the relative size of a measure's measurement error (Lexell and Downham, 2005). It is calculated as:

$$\begin{array}{l} SEMeas\%=\frac{SEMeas}{mean}*100 \end{array}$$

We use it here solely to inspect the relative noisiness of various measures.

#### Smallest detectable change (SDC)

The SEMeas is used to estimate the SDC for the measure (Beckerman et al., 2001). Unlike the SEMeas, which estimates the potential scatter of repeated measurements, the SDC estimates amount of change an observation would need to exceed that expected scatter. The SDC is the smallest change in score that, with some degree of certainty, can be declared a real change above the measurement error (Beckerman et al., 2001; Terwee et al., 2007). If a measurement changes by less than the SDC, it is assumed to be measurement noise. Of note, “real change” does not imply that the change validly reflects a changing construct or is clinically meaningful, issues we discuss later. SDC is also known as the smallest real difference, true change, minimal detectable difference and minimal detectable change, and is conceptually similar to the limits of agreement (Bland and Altman, 1986). The SDC can determined for an individual (SDC_indiv_) or a group (SDC_group_).

The SDC for an individual is the 95% confidence interval of the SEMeas of the change scores (Schuck and Zwingmann, 2003; Terwee et al., 2007), and is calculated as:

$$\begin{array}{l} {SDC}_{indiv}=SEMeas*\sqrt{2}*1.96 \end{array}$$

where $\sqrt{2}$ accounts for the variances associated with 2 independent sessions (e.g., pre, post) used to calculate the change score (see derivation in Schuck and Zwingmann, 2003), and 1.96 represents a 95% confidence interval, assuming normally distributed scores. The SDC for a group is based on the individual SDC (Terwee et al., 2007; de Vet et al., 2011), and is calculated as:

$$\begin{array}{l} {SDC}_{group}=\frac{SDC_{indiv}}{\sqrt{n}} \end{array}$$

Like the SEMeas, the SDC is stable across the spectrum of measurements for a measure, as long as change scores are not heteroscedastic. In other words, once assured of change score homoscedasticity, the absolute SDC can be applied to measurements regardless of where they lie in the spectrum.

#### Reliability_MP_: intraclass correlation coefficient (ICC)

The decomposed variances were also used to estimate reliability_MP_. Reliability_MP_ assesses how well the measure can tell subjects apart despite measurement error (de Vet et al., 2006, 2011; Terwee et al., 2007; Streiner and Norman, 2008). It gauges the consistency of an individual's position relative to others in a group assessed at test-retest or between raters (Weir, 2005; Streiner and Norman, 2008). Reliability_MP_ is estimated using the Intraclass Correlation Coefficient (ICC), and can be calculated using several different formulae, depending on the intended interpretation and application of the results (Shrout and Fleiss, 1979; McGraw and Wong, 1996). Generally speaking, the ICC is the proportion of between-subject variance to all sources of variance (Shrout and Fleiss, 1979; Mokkink et al., 2010). For our study, where test-retest measurements were taken on separate days with a single TMS operator, the ICC is calculated as:

$$\begin{array}{l} {ICC}_{agreement}=\frac{\sigma_{subjects}^{2}}{\sigma_{subjects}^{2}+\sigma_{day}^{2}+\sigma_{residual}^{2}} \end{array}$$

This formula is identical to ICC (2, k) or (A,k) (Shrout and Fleiss, 1979; McGraw and Wong, 1996; Streiner and Norman, 2008).

ICC values range from 0 to 1, with 1 being highest possible reliability_MP_. Guidelines for the interpretation of ICC suggest that a value >0.70 is acceptable reliability_MP_, but acknowledge that this threshold was arbitrarily demarcated (Portney and Watkins, 2009). Because of the way the ICC is calculated, a defining feature is the influence of sample heterogeneity—the larger the between-subject variance, $\sigma_{subjects}^{2}$, the higher the ICC. This is not to say that measurement error (i.e., all other sources of variance) has *no* influence on reliability_MP_, just that the between-subject variance has relatively more (de Vet et al., 2006). Given the interpretation of reliability_MP_, this calculation makes sense: the wider the dispersion of the subjects, the better the measure will be at telling subjects apart, almost regardless of the measurement noise within-subject. Conversely, if the sample's subjects are narrowly distributed, the instrument will have difficulty telling subjects apart, even with a small measurement error (de Vet et al., 2006). As such, reliability_MP_ is not an intrinsic property of the measure, but is rather the combined property of the measure and the subject sample in which it was tested (Tighe et al., 2010).

### Statistical methods

Bland-Altman plots were used to inspect the homoscedasticity of change scores for the measures made on each hemisphere in each group (Bland and Altman, 1986). Differences between morning and afternoon sessions for each subject were plotted against the mean score of the two sessions. Heteroscedasticity, determined by visual inspection, was noted for RC slope and LICI. For these measures, group comparisons and assessment of reliably can be made, but only after appropriately transforming the observations. The most common transformation in such a situation is the log transform; thereafter, comparative differences, SEMeas, SDC, and ICC can still be computed and reported, but are interpreted for the logarithmically transformed values of the measure. Also, because log-transformed data are no longer on a ratio scale, we do not calculate ratios such as SEMeas% for LICI and RC slope.

For continuous demographic data, group means and standard deviations were obtained by averaging outcomes across subjects. For TMS measures, group means and standard deviations for each hemisphere were obtained by first averaging each subject's measurements for the 4 sessions, then averaging across the 20–21 subjects within group.

For the comparison of categorical demographic data, Fisher's exact test was used; for continuous demographic data, a one-way ANOVA with *post-hoc* group-wise comparisons was used, with a Bonferroni adjustment for multiple comparisons between subject groups.

For TMS data, mean outcomes from each hemisphere were compared within and across groups. The outcomes of the healthy older hemispheres were averaged to create a “healthy control” hemisphere, against which the lesioned and non-lesioned hemispheres of stroke subjects were separately compared. For the heteroscedastic measures (LICI, RC slope), comparisons were performed on the logarithmically transformed data. For TMS data, a one-way ANOVA with *post-hoc* group-wise comparisons was used, followed by a Bonferroni adjustment for multiple comparisons. Corrections were made for comparisons between hemispheres within-group (e.g., lesioned chronic vs. non-lesioned chronic), type of hemisphere in one stroke group vs. same type of hemisphere in the other stroke group (e.g., lesioned chronic vs. lesioned subacute), and hemisphere in one stroke group vs. the “healthy control” hemisphere (e.g., lesioned chronic vs. healthy control). Significance was set at *p* < 0.05.

Confidence intervals were obtained for the SEMeas, SDC, and ICC for each measure using a standard bootstrapping resampling algorithm. Standard errors of the reliability estimates rely very heavily on properties of the assumed normal distribution, while bootstrap-based intervals are non-parametic and are valid for any distribution (Efron and Tibshirani, 1993).

## Results

### Clinical and psychometric characteristics

Mean gender, handedness, and racial composition were not significantly different across groups (Table 1). Subacute stroke subjects were significantly older than chronic stroke subjects (*p* < 0.01) and trended older than healthy older subjects (*p* = 0.09). Both stroke groups had significantly more comorbidities than healthy older subjects (both *p* < 0.005), but psychoactive medication intake was similar across the groups. The paretic FDI of subacute and chronic stroke subjects was significantly weaker than right FDI of healthy older subjects (both *p* < 0.01) on the MRC scale but not by MVC. The side and the location of the strokes were not significantly different between subacute and chronic subjects. As expected, significantly more days since the stroke had elapsed in chronic than in subacute stroke subjects (*p* < 0.01).

**Table 1:** **Clinical characteristics**.

**Groups**	**Healthy older**	**Subacute stroke**	**Chronic stroke**
N	21	20	21
Age (years)	64.7 ± 10.1	72.2 ± 12.7	62.0 ± 9.2
Gender	10M: 11F	11M: 9F	15M: 6F
Handedness	0.9 ± 0.4	0.9 ± 0.5	0.8 ± 0.5
Race (White: Black: Asian)	16W: 3B: 1A	15W: 5B: 1A	16W: 3B: 2A
Number of comorbidities	1.3 ± 1.4	3.1 ± 1.3	2.8 ± 1.3
Psychoactive meds (% taking)	20.0%	20.0%	23.8%
FDI abduction strength (MRC)	5.0 ± 0.0	4.4 ± 0.6	4.0 ± 1.0
Lateral pinch strength (kg)	4.66 ± 2.20	4.09 ± 1.33	4.44 ± 2.00
Lesion type (Subcortical: Mixed)	–	11S: 9M	12S: 9M
Lesioned hemisphere (Left: Right)	–	9L: 11R	15L: 6R
Time since stroke (days)	–	17.4 ± 9.8	2617.9 ± 3166.1

Age, Edinburgh handedness score, number of comorbidities, FDI strength, MVC, and time since stroke are mean ± SD. The proportion of the group taking standing psychoactive medications is listed. FDI MRC score and pinch strength are shown for the right hand of healthy subjects and the paretic hand of stroke subjects.

Self-reported levels of alertness, excitement to participate, and caffeine intake were not significantly different across groups (Table 2). Chronic stroke subjects slept significantly more hours than healthy older subjects (*p* < 0.05). Chronic stroke subjects also spent significantly less time exercising in the hours before the testing session than subacute stroke or healthy older subjects (both *p* < 0.05).

**Table 2:** **Psychometric data**.

**Groups**	**Healthy older**	**Subacute stroke**	**Chronic stroke**
Alertness	7.7 ± 1.3	7.9 ± 1.3	7.6 ± 1.1
Excitement	8.1 ± 1.1	8.1 ± 1.9	8.1 ± 1.7
Sleep duration (h)	6.4 ± 1.5	7.0 ± 1.5	7.6 ± 1.5
Caffeine intake (cups)	0.7 ± 0.6	0.4 ± 0.4	0.7 ± 0.5
Exercise duration (min)	23.6 ± 15.4	23.0 ± 39.0	12.4 ± 10.8

All self-reported outcomes are mean ± SD. Alertness and excitement are on a scale of 1–10 (10 is maximum). Sleep duration includes daytime naps.

### Missing and excluded TMS data

An accidental recording failure occurred for a single LICI and TS_MEP_ set in a chronic stroke subject and for a single SICI_80_, ICF_80_, and TS_MEP_ set for a healthy older subject (0.6% data missing from each group); all other data collections were complete. Due to an active pre-stimulus EMG baseline, 3.1% of single trials from healthy older, 6.3% from subacute, and 3.7% from chronic stroke subjects were removed. Inappropriate model fits prompted removal of 5.9% of healthy older, 9.4% of subacute stroke, and 12.7% of chronic stroke RC estimations.

### TMS measurements within and across groups

We averaged hemispheric data and compared within and across groups to verify similarity to measurements previously documented in the field (Table 3). Though RC slope and LICI are transformed for comparison of their means and estimation of their reliability measurement properties, means of their untransformed data are shown to facilitate comparison to extant results.

**Table 3:** **TMS measures**.

**Groups**	**Healthy older**		**Subacute stroke**		**Chronic stroke**	
**Hemisphere**	**Left**	**Right**	**Lesioned**	**Non-lesioned**	**Lesioned**	**Non-lesioned**
rMT (%MSO)	48.67 ± 9.54	50.35 ± 8.49	51.15 ± 12.65	47.22 ± 10.46	55.89 ± 10.62^*^	46.14 ± 7.39^*^
TS_MSO_ (%MSO)	67.52 ± 12.08	65.42 ± 10.52	67.48 ± 17.76	64.41 ± 17.46	77.44 ± 13.34^*^^‡^	62.90 ± 11.56^*^
TS_MEP_ (mV)	1.34 ± 0.62	1.31 ± 0.43	0.94 ± 0.67	1.25 ± 0.42	0.72 ± 0.67^*^^‡^	1.29 ± 0.44^*^
RC slope (mV/%MSO)	0.38 ± 0.37	0.50 ± 0.40	0.43 ± 0.70	0.28 ± 0.17	0.68 ± 1.28	0.46 ± 0.54
RC S_50_ (%MSO)	61.56 ± 13.35	61.46 ± 12.07	61.07 ± 13.44	61.57 ± 14.21	61.41 ± 12.04	60.38 ± 10.53
RC plateau (mV)	2.20 ± 1.30	2.42 ± 1.38	1.83 ± 1.56	2.64 ± 1.35	1.08 ± 1.23^*^^‡^	2.06 ± 0.98^*^
SICI_60_	0.76 ± 0.31	0.72 ± 0.26	0.82 ± 0.26	0.74 ± 0.33	0.82 ± 0.22	0.74 ± 0.28
SICI_80_	0.41 ± 0.22	0.39 ± 0.18	0.64 ± 0.32^‡^	0.60 ± 0.30^†^	0.61 ± 0.26^‡^	0.54 ± 0.36
ICF_60_	1.20 ± 0.18	1.22 ± 0.24	1.32 ± 0.40	1.21 ± 0.24	1.31 ± 0.42	1.12 ± 0.25
ICF_80_	1.39 ± 0.24	1.34 ± 0.31	1.55 ± 0.77	1.30 ± 0.39	1.60 ± 0.70	1.39 ± 0.36
LICI	0.29 ± 0.38	0.23 ± 0.32	0.12 ± 0.18	0.25 ± 0.36	0.36 ± 0.39	0.22 ± 0.29

Measurements are mean ± SD from the 4 sessions. All paired-pulse measurements are decimal percentage of the unconditioned stimulus.

* Significant within-group interhemispheric difference.

† Significant difference between the non-lesioned hemisphere and healthy control hemisphere.

‡ Significant difference between lesioned hemisphere and healthy control hemisphere.

Within healthy older and subacute stroke groups, outcomes were not significantly different between hemispheres. In chronic stroke, compared to the non-lesioned hemisphere, the lesioned hemisphere had a significantly lower RC plateau (*p* < 0.05) and TS_MEP_ (*p* < 0.01); it also had a significantly higher rMT (*p* < 0.005) and TS_MSO_ (*p* < 0.005). As outcomes were not significantly different across hemispheres in healthy older subjects, we combined their data to create a “healthy control” hemisphere against which the lesioned and non-lesioned hemispheres of stroke subjects were compared.

Compared to the healthy control hemisphere, SICI_80_ was reduced in the lesioned hemispheres of subacute and chronic stroke (both *p* < 0.05; higher decimal fraction connoting disinhibition) and in the non-lesioned hemisphere of subacute stroke only (*p* < 0.05). Compared to healthy control hemisphere, RC plateau and TS_MEP_ were decreased (both *p* < 0.01), and TS_MSO_ was increased (*p* < 0.05) in the lesioned hemisphere of chronic stroke. Comparing stroke groups, outcomes were not significantly different between the two non-lesioned hemispheres or between the two lesioned hemispheres.

### Reliability measurement properties

SEMeas and SDC_indiv_ are shown for each group's hemisphere and outcome (Table 4). For untransformed measures, SEMeas and SDC_indiv_ values are reported in units particular to the measure: rMT, TS_MSO_, and RC S_50_ are in %MSO; RC plateau is in mV; and SICI and ICF are in the decimal fraction of TS_MEP_. As an example of interpretation, take for instance the left hemisphere of the healthy older adult. Measurement error was 1.72%MSO points around an observed rMT, and a stimulator intensity change of at least 4.77%MSO points would be necessary to exceed measurement noise in an individual. Similarly, measurement error was a 0.22 decimal fraction of TS around an observed ICF_80_ value, and would have to change by at least 0.62 to be declared a real change exceeding noise in an individual.

**Table 4:** **Standard error of the measurement (SEMeas) and smallest detectable change for individuals (SDC_indiv_) with lower and upper 95% confidence intervals in parentheses**.

**Groups**	**Healthy older**				**Subacute stroke**				**Chronic stroke**			
**Hemisphere**	**Left**		**Right**		**Lesioned**		**Non-lesioned**		**Lesioned**		**Non-lesioned**	
	**SEMeas**	**SDC_indiv_**	**SEMeas**	**SDC_indiv_**	**SEMeas**	**SDC_indiv_**	**SEMeas**	**SDC_indiv_**	**SEMeas**	**SDC_indiv_**	**SEMeas**	**SDC_indiv_**
rMT	1.72(0.58, 2.68)	4.77(1.61, 7.43)	1.29(0.84, 1.75)	3.58(2.32, 4.85)	2.41(0.99, 3.64)	6.67(2.75, 10.08)	1.04(0.70, 1.32)	2.88(1.94, 3.67)	2.07(1.12, 2.68)	5.75(3.11, 7.42)	1.17(0.85, 1.36)	3.24(2.36, 3.78)
TS_MSO_	1.60(0.85, 2.17)	4.42(2.36, 6.00)	1.95(1.22, 2.55)	5.41(3.38, 7.08)	1.92(0.95, 2.49)	5.33(2.63, 6.91)	2.11(1.20, 3.06)	5.85(3.33, 8.47)	1.57(1.26, 2.79)	4.36(3.50, 7.74)	2.65(1.57, 3.34)	7.34(4.35, 9.25)
Ln(RC slope)	0.66(0.43, 0.91)	1.83(1.19, 2.53)	0.76(0.59, 1.03)	2.10(1.64, 2.87)	0.48(0.29, 0.61)	1.33(0.80, 1.68)	0.39(0.27, 0.52)	1.08(0.74, 1.45)	0.84(0.51, 1.26)	2.32(1.41, 3.50)	0.66(0.40, 0.98)	1.83(1.12, 2.72)
RC S_50_	4.12(2.54, 5.30)	11.42(7.03, 14.69)	3.52(2.03, 4.51)	9.76(5.63, 12.49)	5.40(3.22, 7.19)	14.98(8.93, 19.92)	4.18(2.84, 5.20)	11.58(7.88, 14.41)	4.31(2.58, 6.41)	11.95(7.16, 17.76)	5.18(2.47, 7.73)	14.37(6.85, 21.41)
RC plateau	0.42(0.29, 0.51)	1.16(0.81, 1.40)	0.61(0.30, 0.87)	1.68(0.84, 2.42)	0.38(0.14, 0.57)	1.06(0.38, 1.58)	0.60(0.37, 0.70)	1.66(1.03, 1.95)	0.19(0.12, 0.27)	0.54(0.34, 0.76)	0.59(0.35, 0.81)	1.63(0.97, 2.24)
SICI_60_	0.18(0.13, 0.22)	0.51(0.35, 0.62)	0.22(0.11, 0.31)	0.60(0.28, 0.87)	0.19(0.11, 0.28)	0.53(0.30, 0.77)	0.18(0.11, 0.24)	0.50(0.31, 0.67)	0.21(0.11, 0.30)	0.58(0.30, 0.82)	0.19(0.12, 0.23)	0.52(0.34, 0.63)
SICI_80_	0.11(0.08, 0.13)	0.31(0.22, 0.37)	0.16(0.11, 0.19)	0.44(0.30, 0.53)	0.17(0.08, 0.22)	0.46(0.26, 0.60)	0.17(0.11, 0.23)	0.48(0.31, 0.63)	0.21(0.13, 0.27)	0.59(0.36, 0.75)	0.13(0.08, 0.15)	0.35(0.24, 0.42)
ICF_60_	0.21(0.16, 0.28)	0.59(0.45, 0.77)	0.28(0.14, 0.44)	0.79(0.39, 1.21)	0.33(0.14, 0.50)	0.90(0.40, 1.38)	0.22(0.15, 0.27)	0.61(0.40, 0.74)	0.51(0.19, 0.86)	1.42(0.53, 2.38)	0.27(0.20, 0.37)	0.74(0.54, 1.02)
ICF_80_	0.22(0.18, 0.29)	0.62(0.50, 0.80)	0.36(0.28, 0.50)	1.00(0.77, 1.40)	0.59(0.16, 1.06)	1.63(0.44, 2.93)	0.22(0.14, 0.27)	0.60(0.39, 0.74)	0.78(0.48, 1.29)	2.17(1.33, 3.58)	0.34(0.22, 0.43)	0.95(0.62, 1.20)
Ln(LICI)	0.55(0.37, 0.71)	1.54(1.01, 1.98)	0.54(0.32, 0.69)	1.50(0.88, 1.91)	0.42(0.26, 0.50)	1.16(0.71, 1.38)	0.57(0.39, 0.78)	1.59(1.08, 2.17)	0.60(0.29, 0.87)	1.65(0.79, 2.42)	0.53(0.35, 0.63)	1.47(0. 97, 1.74)

SEMeas is the measurement error of the measure, whereas SDC_indiv_ delimits the smallest delta that is considered a real measurement change for an individual. SEMeas and SDC_indiv_are in units particular to the measure for non-transformed data: rMT, TS_MSO_, and RC S_50_ are %MSO; RC plateau is mV; SICI and ICF are in decimal fraction of TS. The SEMeas and SDC for ln(RC slope) and ln(LICI) are dimensionless, but are based on original units of mV/%MSO (slope) and decimal fraction of TS (LICI). To calculate SDC_group_ for a group of size n, divide SDC_indiv_ by $\sqrt{n}$. SDC_group_ is the smallest delta that is considered a real measurement change for a group of size n.

For transformed measures, SEMeas and SDC_indiv_ values are unitless, but are based on data in their original units: mV/%MSO for RC slope and decimal fraction of TS_MEP_ for LICI. Staying with the left hemisphere of the healthy older adult, measurement error is 0.66 around the observed ln(RC slope) value, and a difference of at least 1.83 between two observed ln(RC slope) values would be needed to exceed measurement noise in an individual.

**Figure 1:**
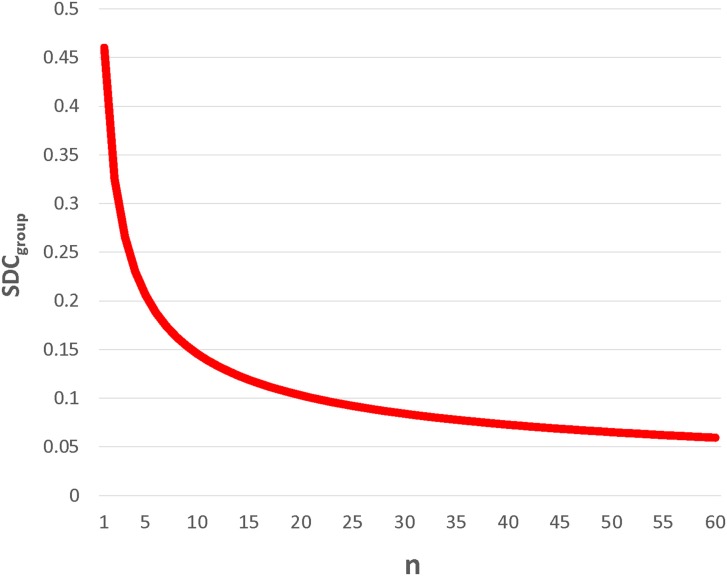
**SDC**_group_ **decreases dramatically with increasing n**. This example, using SICI_80_ in lesioned hemisphere of subacute stroke, demonstrates for that for an individual, the SDC is quite large, requiring a change greater than 0.45 in SICI_80_ in the lesioned hemisphere to be deemed real. By calculating SDC_group_ (= SDC_indiv_/$\sqrt{\text{n}}$), it is apparent that even with modest sample sizes of 10–20, changes exceeding measurement error could be conceivably detected, and the measure would be considered reliable.

Importantly, SDC_indiv_ is provided to enable future investigators to generate SDC_group_ for their samples of size *n*. Given that measurement error changes with context, it is imperative that investigators calculate the SDC_group_ pertaining to each subject type, hemisphere, and sample size. As would be expected, SDC_group_ shrinks dramatically as group size increases. A graphical example is given for SICI_80_ in the lesioned hemisphere of subacute stroke subjects (Figure 1). For SICI_80_, changes greater than SDC_indiv_ = 0.46 would be required to exceed measurement error for an individual, but as n increases beyond 19, SDC_group_ ≤ 0.10.

Across all measures and all groups, SDC_indiv_ were sizable. SEMeas% provides an impression of the relative noisiness of a measure; because transformed data are no longer on a ratio scale, LICI and RC slope are excluded (Table 5). Others have used SEMeas% < 10% as a cutoff for high measurement stability, though this threshold is arbitrary (Flansbjer et al., 2005). From Table 5, the measures with the lowest relative measurement error were rMT (2.2–4.7%), TS_MSO_ (2.0–4.7%), and RC S_50_ (5.7–8.9%), with RC plateau (18.1–28.4%), SICI (24.3–40.8%), and ICF (16.1–48.9%) having moderate measurement error. In general, measures were noisier in the right compared to the left hemisphere of healthy older subjects, and were marginally noisier in the lesioned compared to the non-lesioned hemispheres of subacute and chronic stroke subjects. SICI and ICF were slightly noisier when obtained with a CS of 80% rMT than with 60% rMT.

**Table 5:** **Relative measurement error**.

**Groups**	**Healthy older**		**Subacute stroke**		**Chronic stroke**	
**Hemisphere**	**Left**	**Right**	**Lesioned**	**Non-lesioned**	**Lesioned**	**Non-lesioned**
rMT	3.53	2.56	4.70	2.20	3.71	2.54
TS_MSO_	2.36	2.98	2.85	3.28	2.03	4.21
RC S_50_	6.69	5.73	8.85	6.78	7.02	8.58
RC plateau	18.96	25.05	20.85	22.63	18.07	28.44
SICI_60_	24.28	30.18	23.49	24.49	25.68	25.25
SICI_80_	26.81	40.83	25.91	29.12	34.76	23.22
ICF_60_	17.71	23.17	24.75	18.12	39.22	23.71
ICF_80_	16.13	26.70	38.06	16.70	48.90	24.54

SEMeas% is measurement error relative to the mean measurement of the outcome. It is used to inspect the relative noisiness of the measure. Measures with SEMeas% < 10% may imply better measurement stability. RC slope and LICI are excluded because transformed data are no longer on ratio scale.

Reliability_MP_ is shown for each group's hemisphere and outcome (Table 6). The ICC quantifies reliability_MP_, the measure's ability to distinguish between subjects in a sample. As the ICCs of RC slope and LICI were based on logarithmically transformed data, their ICCs comment on how well the transformed values of the measure can discriminate between subjects in a sample. ICC > 0.70 is generally considered good reliability_MP_ (Portney and Watkins, 2009). By this convention, rMT, TS_MSO_,RC S_50_,RC plateau, and ln(LICI) had generally good reliability_MP_, whereas RC slope and all SICI and ICF measures had generally poorer reliability_MP_. SICI and ICF had higher ICCs when obtained with a CS of 80% rMT compared with 60% rMT. For the most part, measures taken in healthy older subjects had marginally higher reliability_MP_ in the left than the right hemisphere. In subacute stroke subjects, measures from the lesioned hemisphere had slightly higher reliability_MP_ than the non-lesioned hemisphere; the opposite occurred in chronic stroke subjects. Generally measures taken in subacute stroke subjects had the highest reliability_MP_.

**Table 6:** **Intraclass correlation coefficient (ICC) with lower and upper 95% confidence intervals in parentheses**.

**Groups**	**Healthy older**		**Subacute stroke**		**Chronic stroke**	
**Hemisphere**	**Left**	**Right**	**Lesioned**	**Non-lesioned**	**Lesioned**	**Non-lesioned**
rMT	0.97 (0.90,0.99)	0.98 (0.96,0.99)	0.96 (0.91,0.99)	0.99 (0.98,0.99)	0.96 (0.93,0.98)	0.98 (0.96,0.99)
TS_MSO_	0.98 (0.96,0.99)	0.97 (0.94,0.99)	0.99 (0.97,0.99)	0.99 (0.96,0.99)	0.99 (0.96,0.99)	0.95 (0.91,0.98)
Ln(RC slope)	0.03 (0,0.51)	0.07 (0,0.70)	0.70 (0.35,0.84)	0.53 (0.26,0.77)	0.18 (0,0.86)	0.23 (0,0.71)
RC S_50_	0.91 (0.80,0.95)	0.92 (0.82,0.96)	0.85 (0.72,0.94)	0.92 (0.85,0.96)	0.88 (0.77,0.97)	0.78 (0.49,0.92)
RC plateau	0.90 (0.82,0.94)	0.82 (0.62,0.94)	0.94 (0.87,0.98)	0.82 (0.66,0.90)	0.98 (0.95,0.99)	0.71 (0.55,0.84)
SICI_60_	0.71 (0.52,0.83)	0.51 (0.31,0.70)	0.60 (0.33,0.82)	0.74 (0.57,0.90)	0.33 (0,0.78)	0.64 (0.28,0.80)
SICI_80_	0.78 (0.62,0.86)	0.44 (0.01,0.63)	0.77 (0.57,0.88)	0.71 (0.47,0.86)	0.49 (0.20,0.72)	0.88 (0.77,0.92)
ICF_60_	0.04 (0,0.59)	0.23 (0,0.73)	0.48 (0,0.85)	0.43 (0.03,0.66)	0.15 (0,0.81)	0.00 (0,0.69)
ICF_80_	0.45 (0.17,0.75)	0.32 (0,0.79)	0.56 (0.30,0.94)	0.73 (0.53,0.86)	0.16 (0,0.92)	0.38 (0,0.70)
Ln(LICI)	0.88 (0.76,0.94)	0.88 (0.76,0.94)	0.89 (0.81,0.95)	0.86 (0.73,0.94)	0.87 (0.75,0.97)	0.89 (0.79,0.93)

ICC quantifies reliability_MP_, which is how well a measure can distinguish between subjects in a sample. ICC > 0.70 is considered good reliability_MP_.

### Diagnostics

To insure that variability did not change over the course of the study (e.g., because drift in operator technique) we inspected the variances of each measure from the first and last 5 subjects of the three groups. We did not see a systematic pattern of variance change over time. Given the fixed testing order for the measures, we also inspected order effects on variance. We compared within-subjects variance of TS_MEP_ obtained with same TS_MSO_, recorded earlier and later in the session (i.e., TS_MEP_ used for SICI_60_/ICF_60_ vs. LICI). We found no systematic pattern of change in TS_MEP_ variance in either hemisphere in any subject group.

## Discussion

In the present study, we characterized the reliability of common TMS measures in healthy older subjects and stroke subjects, using variances estimated from a four-session test-retest paradigm. We distinguished between reliability measurement properties that inform the two main uses of TMS: (1) evaluation of change in subjects, for which measurement error must be considered; and (2) discrimination between subjects within a sample, for which reliability_MP_ must be considered. We additionally used the measurement error to generate the SDC, which assists investigators in deciding, for a given sample size, whether an observed change is sufficiently large to exceed measurement error.

We found that SDCs for individuals were universally large across all TMS measures, precluding reasonable single-subject evaluative use; therefore these TMS measures *should not* be used as biomarkers for assessing individual change. For these same TMS measures, SDCs become sufficiently low in modest sample sizes to justify their evaluative use; therefore these measures *can* be used to detect group-level changes. Reliability_MP_ was highly variable for many measures in our subject groups. However, for samples with similar measurement distributions as ours, rMT, TS_MSO_, RC S_50_, RC plateau, and ln(LICI) could be used for discriminatory purposes, for example, for patient staging or diagnosis.

### TMS measurement outcomes in healthy aging and stroke subjects

Our TMS technique produced measurements that largely replicate the outcomes of other neurophysiology studies conducted on the FDI of healthy older subjects (Bütefisch et al., 2003, 2008; Delvaux et al., 2003; Werhahn et al., 2003; Fridman et al., 2004; Swayne et al., 2008; Takechi et al., 2014), subacute stroke subjects (Bütefisch et al., 2003, 2008; Delvaux et al., 2003; Liepert et al., 2005; Swayne et al., 2008; Prashantha et al., 2013), and chronic stroke subjects (Delvaux et al., 2003; Werhahn et al., 2003; Fridman et al., 2004; Liepert, 2006; Swayne et al., 2008; Takechi et al., 2014).

One exception was a relatively lower rMT observed in the lesioned hemisphere of our subacute stroke subjects compared to what some have recorded. This difference could arise from a common practice of assigning an MSO of 100% when there is an absent motor response (Bütefisch et al., 2008; Swayne et al., 2008; Takechi et al., 2014). This approach inflates group averages (Heald et al., 1993), and removal of non-responders from the group aggregate reveals a reduction in the group rMT (Bütefisch et al., 2008). Another source of dissimilarity may arise from equipment differences: absolute magnetic field strength associated with %MSO varies with stimulator brand, coil shape, and Bistim vs. single-stimulator mode for the Magstim device. A lower rMT in the lesioned hemisphere may also indicate better preserved corticospinal tract integrity in our patient sample, as may be inferred from their reasonably high level of function.

Comparing the magnitude of SICIs elicited by different CS intensities, we did not appreciate an inhibitory nadir with a CS at 60% rMT (SICI_60_), as has been observed in the non-lesioned hemisphere (Bütefisch et al., 2003, 2008) and lesioned hemisphere (Bütefisch et al., 2008) of subacute stroke subjects. Rather, inhibition was stronger with a CS at 80% rMT (SICI_80_) in all subject groups, similar to that seen in healthy younger subjects (Kujirai et al., 1993). Differences may result from our use of higher TS MEP amplitudes, as per the original SICI paradigm (Kujirai et al., 1993); perhaps there is a unique interaction between CS intensity and TS amplitude in stroke subjects, given each affects apparent SICI (Kujirai et al., 1993; Sanger et al., 2001). In keeping with other observations, SICI_80_ was reduced in both hemispheres of acute stroke subjects (Liepert et al., 2000b; Bütefisch et al., 2003, 2008; Takechi et al., 2014) and in the lesioned hemisphere of chronic stroke subjects (Swayne et al., 2008) relative to healthy controls.

In summary, our TMS measurements are in line with the majority of previous studies in healthy older and stroke subjects, including observed similarities and differences between the healthy and stroke groups. As with any study, it is possible that despite correcting for multiple comparisons, our comparative findings may have arisen by chance alone. However, our replication of past observations supports the generalization of our reliability results to conventional TMS paradigms.

### Reliability measurement properties

The following discussion of reliability results is restricted to previous studies focusing on FDI neurophysiology in older healthy subjects and stroke subjects. TMS measures do not behave the same with age (Rossini et al., 1992; Peinemann et al., 2001; McGinley et al., 2010) or in different muscle representations (Brasil-Neto et al., 1992; Malcolm et al., 2006; Menon et al., 2014), and therefore we do not assume that their variances or derived reliability measurement properties are similar.

#### Measurement error and smallest detectable change

Two previous reliability studies estimated the SEMeas of TMS measures in FDI of chronic stroke subjects (Koski et al., 2007; Liu and Au-Yeung, 2014), although differences in paradigm, analysis, and measures preclude comparison to the first study (Koski et al., 2007). Our findings were comparable to work by Liu and colleagues (Liu and Au-Yeung, 2014), who found rMT SEMeas in chronic stroke subjects was 1.9 MSO points in the lesioned hemisphere and 2.02 in the non-lesioned hemisphere for rMT, similar to our 2.07 and 1.17, respectively. To our knowledge, SEMeas has not been previously reported for other TMS measures in the FDI of chronic stroke, or for any measure in the FDI of subacute stroke or healthy older subjects. SDC has not been estimated for any measures in the FDI of healthy older adults or stroke subjects. A recent study used the Limits of Agreement (LOA), similar in concept to the SDC, to estimate test-retest reliability of total motor conduction time in subacute stroke subjects (Hoonhorst et al., 2014).

Just how noisy are the TMS measures? Are the SEMeas and SDC small enough to encourage the use of the measure within individuals, i.e., as a biomarker in clinical practice? There are no universally accepted norms for an acceptably low SEMeas or SDC. One approach is to use a relative measurement error (SEMeas%) < 10% as a cutoff for high measurement stability (Flansbjer et al., 2005). SEMeas% in our study ranged from very low (e.g., 2.2% for subacute non-lesioned rMT) to moderately high (e.g., 48.9% for chronic lesioned ICF_80_). The measures with generally the lowest relative measurement error were rMT, TS_MSO_, and RC S_50_, with the highest being SICI and ICF. In general, the relative measurement error was marginally lower in the left healthy older hemisphere and in the non-lesioned stroke hemispheres, suggesting a possible role for hemispheric dominance and non-pathology contributing to measurement stability.

In reality, however, whether a measure is sufficiently stable for practical individual use is determined by the amount of change that one could reasonably expect in the measurements with a true clinical state change. Take by analogy the hematocrit, the percent of red blood cells in a sample. The hematocrit SDC is approximately 3% points at most institutions; that is, the hematocrit can change by 3 points and still be considered measurement noise. Clinicians tolerate this SDC because a real change in clinical state generally produces a hematocrit change exceeding 3 points. If instead the hematocrit SDC was 20 points, measurement error may produce a retest value that would be interpreted as a clinical change in a stable patient; in this case, hematocrit would be unreliable for measuring change in the individual. Thus, an overall sense of expected real change in the individual or group is helpful for determining whether the measure has a sufficiently low SDC to be considered a viable biomarker.

Across groups for most TMS measures, SDC_indiv_ exceeds what is likely to be observed in an individual changing due to a standard intervention or a clinical state change. Take the left hemisphere of a healthy subject for example: after intervention, it would be unusual to observe more than a 4.4%MSO adjustment to reproduce TS_MEP_, a RC plateau change greater than 1.1 mV, a SICI_80_ change greater than 0.31, a ICF_80_ change greater than 0.62, or an ln(LICI) change greater than 1.54 (equivalent to a change of *e*^1.54^, or 466%, in LICI); the other hemisphere and groups generally require even larger changes (Table 4). We therefore advise against using TMS measures to track changes within the individual; SDC_indiv_ are too large to be used as individual biomarkers for the changes that can be reasonably expected with our current interventions. (The exception, of course, is if an intervention is particularly potent and induces marked change.)

With increasing *n*, the SDC_group_ becomes smaller, i.e., smaller average measurement changes are required to exceed measurement noise. One may use the SDC_group_ to ascertain whether a change exceeding measurement error has occurred in collected data. Inferential statistics (i.e., *t*-tests to test for the significance of the change) are complimentary, giving concordant information if the measurement variances of our group and the compared sample are similar. If an observed change exceeds SDC_group_ and is statistically significant, it is likely real measurement change; if the observed change does not exceed SDC_group_ and is non-significant, it is likely measurement noise. Given that our variances were derived from a large number of observations, to match the precision of our estimates, the compared sample would also need to be large.

Variances are prone to be dissimilar with small sample sizes. In scenarios where the change exceeds the SDC_group_ but it is not significant, or vice-versa, the discordance is explained by differences in variance estimates of the sample compared to ours. Thus, a non-significant change exceeding the SDC_group_ implies a higher variability in the compared sample, whereas a significant change less than the SDC_group_ implies a lower variability in the compared sample. Higher or lower variability may have arisen through differences in instrument technique or chance in the sample of subjects.

A change in excess of the SDC_group_, even if statistically significant, does not guarantee that it is “true,” i.e., that it reflects real change in the population (Button et al., 2013). It is critically important that the sample be large enough to ensure robust statistical power—the ability to detect a true population-representative effect. A formal power analysis accounts for both measurement error and probability of detecting a significant change. If a study is known to be adequately powered, then when a statistical test is judged significant, there is a good likelihood that the detected change is in fact true. When underpowered studies find significant differences, these are likely detecting effects that are so inflated that they do not represent a true effect (Button et al., 2013). The highly precise constituent variances derived from our SEMeas and ICCs can and should be used for the appropriate power analyses.

Finally, the potential clinical applications of SDC_indiv_ and SDC_group_are important to distinguish. For a given TMS measure, if an intervention tested in a group of subjects induces a measurement change in excess of the corresponding SDC_group_, the intervention is shown to be effective (or at the minimum, to exceed measurement noise) at the group level. However, this does not mean that the same TMS measure can then be used to detect the intervention's effects in an individual. The SDC_indiv_ is substantially larger than SDC_group_, and only by rare chance (< 5%) or by strong interventional potency might the individual show a TMS measurement change exceeding the SDC_indiv_. What this implies clinically is that a TMS measure could be used to detect a change induced by an intervention in a group, but the same measure may not be used to assess individual efficacy. That is, the intervention will need to be given agnostic to individual TMS outcome, because the TMS measure cannot reliably detect change in the single subject.

#### Reliability_MP_

We estimated reliability_MP_ for our TMS measures, to evaluate the potential for using TMS measures to differentiate between subjects for staging or diagnosis. To our knowledge, TMS has not been applied this way in stroke, aside from the combination of a dichotomous presence/absence of an MEP with other clinical and radiographic features to help prognosticate recovery (Stinear et al., 2014). Although several studies have estimated ICC for TMS measures in young adults (Mortifee et al., 1994; Carroll et al., 2001; Kamen, 2004; Malcolm et al., 2006; Christie et al., 2007; Koski et al., 2007; Cacchio et al., 2009, 2011; Wheaton et al., 2009; Hoonhorst et al., 2014; Liu and Au-Yeung, 2014), few have characterized reliability_MP_ in the FDI of older subjects and subjects with stroke. Again we focus on the estimation of reliability_MP_ for measures taken in the FDI of elderly subjects and stroke subjects, because ICCs between intrinsic hand muscles or between hand and forearm muscles are not the same (Malcolm et al., 2006) and are not expected to be similar for young and old adults.

Guidelines suggest that ICCs > 0.70 indicate acceptable reliability_MP_ to distinguish between subjects (Nunnally and Bernstein, 1994). Of those that did investigate reliability_MP_ in subacute and chronic stroke subjects, generally ICCs > 0.70 were noted for motor thresholds, linearly-derived recruitment curve parameters, silent periods, and total motor conduction time (Koski et al., 2007; Hoonhorst et al., 2014; Liu and Au-Yeung, 2014). Our ICCs for rMT in chronic stroke subjects were comparable to those found by Liu and colleagues; importantly for this comparison, their sample's rMT variances were similar to ours. ICCs were 0.97 in the lesioned hemisphere and 0.95 in the non-lesioned hemisphere, very similar to our 0.96 and 0.98, respectively (Liu and Au-Yeung, 2014). To our knowledge, there have been no studies evaluating reliability_MP_ of any TMS measure in FDI of older healthy subjects, or of paired-pulse measures in subacute or chronic stroke subjects.

In our study, ICCs ranged widely across subject groups and outcomes, with rMT and TS_MSO_ having very high ICCs, and ln(RC slope) and ICF generally low ICCs. Measurements taken in subacute stroke subjects tended to have the highest reliability_MP_. These findings are not counterintuitive. The ICC more predominantly reflects the spread of measurements between subjects and less the measurement error within subjects. For example, the relative measurement errors for SICI_60_ in subacute stroke were 23.5% and 24.5% in the lesioned and non-lesioned hemisphere, respectively. Despite these essentially equivalent SEMeas%, ICCs were 0.60 and 0.74, respectively; the higher ICC was simply due to a larger between-subject variance in the latter. It is for this reason that subacute stroke subjects generally have higher ICCs—not because measurement error is markedly lower in subacute stroke, but because the measurements are more widely dispersed across individuals.

Our findings underscore the direct relationship between the magnitude of the between-subject variance and the magnitude of the ICC (Streiner and Norman, 2008), and how a moderately large measurement error can be obscured by an even larger spread of subjects. It is therefore vital for investigators to understand exactly what the ICC means, how it should be applied, and the constraints on its generalizability.

These three issues are rarely discussed in TMS reliability_MP_ studies for various muscles (Mortifee et al., 1994; Carroll et al., 2001; Kamen, 2004; Malcolm et al., 2006; Christie et al., 2007; Koski et al., 2007; Cacchio et al., 2009, 2011; Wheaton et al., 2009; Hoonhorst et al., 2014; Liu and Au-Yeung, 2014). First, nearly all erroneously interpreted high reliability_MP_ to mean low measurement error. Measures with a high ICC were incorrectly endorsed for evaluative use (Mortifee et al., 1994; Carroll et al., 2001; Kamen, 2004; Malcolm et al., 2006; Christie et al., 2007; Koski et al., 2007; Wheaton et al., 2009; Hoonhorst et al., 2014). Second, none explained the practical application of ICC, which is how well the measure can tell subjects apart, not how well it can detect individual or group change over time (Mortifee et al., 1994; Carroll et al., 2001; Kamen, 2004; Christie et al., 2007; Wheaton et al., 2009; Cacchio et al., 2011; Liu and Au-Yeung, 2014). Only one study pointed out the influence of between-subject variability on ICC estimations (Koski et al., 2007). The frequent observation of a lower reliability_MP_ in a non-lesioned hemisphere or in healthy subjects speaks more to measurement homogeneity, not poor paradigmatic quality—an issue rarely raised.

Finally, no prior TMS reliability_MP_ study has advised that ICCs should be generalized only to future samples with similar measurement spread. Reliability_MP_ is not an intrinsic characteristic of a measurement instrument, but is rather born from the instrument and the sample (Streiner and Norman, 2008). As ICCs tend to be a function of *who* is being measured, special attention needs to be paid to the sample's measurement distribution. A high ICC estimated from a sample with widely dispersed measurements will not be valid for a sample with a narrower distribution. This is not statistical dogmatism, but is simply a reflection of reality: “it *is* more difficult to tell people apart if they are relatively similar (i.e., homogeneous) than if they are very different” (Streiner and Norman, 2008). ICCs must not be assumed to be suitable for new samples without first ensuring that measurement distributions are similar. Our samples' standard deviations (Table 3) are expressly provided for this purpose.

Keeping these points in mind, we found generally high ICCs for rMT, TS_MSO_, RC S_50_, RC plateau, and ln(LICI) in our samples of healthy older adults and stroke subjects. In future samples with similar measurement distributions, these measures could reliably be used to distinguish subjects from one another, i.e., for staging or diagnostic purposes.

### Quality of the study

Studies of test-retest reliability require sufficient sample sizes to estimate variance of change and to make inferences about the relevant population. Sample sizes of 15–50 subjects have been suggested, equating to 30–100 single observations (Fleiss, 1999; Hopkins, 2000; Terwee et al., 2007). In lieu of a larger sample size, we measured each subject in each hemisphere 4 times, each of which was the average of 10 observations, for a total of 80–84 averaged observations per measure per hemisphere. This approach not only gave us a more precise estimate of the overall variance but also allowed us to estimate the various components of the overall variance. We also provide 95% confidence intervals to provide transparency about the level of uncertainty around our estimations.

Studying test-retest reliability assumes that the time between tests is generally long enough to avoid subject learning or carry-over, but short enough that there has not been a clinical change (Terwee et al., 2007). TMS neurophysiology probes are generally believed to be uninfluenced by prior non-modulatory sessions. Neuroplasticity leading to true changes in neurophysiology, particularly for subacute stroke subjects, was of greater concern. We thus chose intervals between tests that were sufficiently short to minimize plasticity-associated neurophysiological change.

Our paradigm called for a fixed testing order, but we do not think this amplified the variability of some measures over others. We noted that LICI had universally large measurement errors and was always the last TMS measure tested in each hemisphere (Table 5). Because direct comparison of LICI and other measures would not disambiguate between timing- and measure-related differences in variance, we inspected the variances of TS_MEP_ obtained with identical TS_MSO_ at separate times over the session. We found no systematic increase or decrease in TS_MEP_ variance in any subject group, suggesting that LICI is inherently noisy.

### Generalizability of the study

It is paramount that any reliability study explicitly delineate the extent to which its results can be generalized. Can our results be extrapolated to other laboratories and operators of TMS? Yes, but with qualification. Reliability is not a unique feature of the measurement instrument, but depends on the sources of variance and the study population (Beckerman et al., 2001). Several key features should thus be considered: the sample, the paradigm, the TMS setup, and the operator.

Measures were obtained solely in the FDI muscles of older healthy subjects and subacute and chronic stroke subjects—and our reliability measurement properties can be used only for the same. We do not assume identical magnitudes in reliability measurement properties in other muscles (Malcolm et al., 2006), in other populations, or in other TMS measures. This work remains to be undertaken.

We purposefully included only stroke subjects whose MEPs could be obtained in both hemispheres, to mirror the partially recovered patient commonly recruited for upper extremity interventional studies. Because of this, most of our subjects had reasonable paretic hand function. Therefore, our samples and their reliability measurement properties are not representative of the universal stroke population. Furthermore, it cannot be assumed that the non-lesioned hemispheres of well-recovered and poorly recovered subjects behave similarly; although both produce MEPs, their neurophysiology and reliability may differ (Manganotti et al., 2008).

Our groups were balanced for race, but all samples were predominantly white. Differences in TMS measurements have been noted for different races (Yi et al., 2014), but differential reliability has not been investigated. The predominance of white subjects in our samples should be kept in mind when extrapolating our results globally. Our samples were also balanced for gender. As our females were postmenopausal and did not take hormone replacement therapy, we do not expect gender-related differences in measurement stability. However, cortical excitability varies with fluctuating ovarian hormone levels in premenopausal subjects (Smith et al., 1999), and measurement error should be separately calculated for test-retest intervals spanning the follicular and luteal phases of the menstrual cycle.

Can the SDC from a short-term assessment (collected over days) be used for long-term test-retest data (collected over weeks or months)? Assuming that subjects are stable on both time scales, the short-term measurement error can be applied to longer-term data (de Vet et al., 2011).

We chose techniques and measures conventionally used in TMS studies. Our TMS setup uses stimulators and recording devices that are commonly used and commercially available. Assuming technique and setups are comparable, variability related to the technology should be similar across laboratories. Ours is the first reliability study in TMS to use neuronavigation to ensure spatial stimulation stability within and between sessions (Schönfeldt-Lecuona et al., 2005), and we strongly recommend the same in studies investigating neurophysiological change. Although marked scalps or swimming caps can direct TMS coil location and yaw, these approaches do not guide coil pitch and roll. It is expected that free-handed positioning of the coil would significantly increase measurement error, as it allows spatial drift (Julkunen et al., 2009) and diminishes the consistency and strength of voltage delivered to a target (Cincotta et al., 2010).

Similarly, electrode positions were outlined directly on the skin to reproduce exact placement over sessions. Given that hand intrinsic muscles have a high density of motor units and large corticospinal representations relative to other muscles (Phillips and Porter, 1977; Brasil-Neto et al., 1992), small variations in electrode placement could lead to the probing of different corticomotoneuronal pathways (Brasil-Neto et al., 1992; Malcolm et al., 2006). To minimize the introduction of additional measurement error by faulty electrode placement, we recommend recording its precise location by marking directly on the skin for short testing intervals, or measuring electrode position relative to bony landmarks for longer intervals. Documenting position with digital photographs or tracings may also be helpful (Butler et al., 2005; Malcolm et al., 2006).

One TMS operator (HS) was used to limit rater-related sources of variability. At the advent of the study, the operator had 4 years of neurophysiology experience in healthy and stroke populations. Over the duration of data collection, variability did not systematically diminish, implying that over some criterion amount of competency, experience may not further influence an investigator's contribution to measurement noise. We assume that our reliability measurement properties would be less applicable to novice TMS investigators. For generalization to all TMS operators, future reliability studies would need to include multiple operators with varying levels of experience.

### Validity and responsiveness

Once reliability has been defined for TMS measures in specific populations, can we freely use the TMS measures to tell us about neurophysiology in these groups? The answer hinges on their validity, how well they actually measure the construct they are purporting to measure (Mokkink et al., 2010), and responsiveness, how well they detect true changes in the construct (Mokkink et al., 2010; de Vet et al., 2011). Like reliability, validity and responsiveness are fundamental quality domains of measurement instruments, and high validity and responsiveness are required to justify their use. Reliability is necessary but not sufficient to determine validity and responsiveness (Portney and Watkins, 2009). Though not the focus of this study, meeting the requirements necessary to deem a TMS measure valid and responsive is a formidable but critical next step for the field.

### Interpretability of measurements in the clinical context

Once the reliability has been established, and a change in an appropriately powered group is observed to exceed the SDC, one must decide whether it has any real clinical meaning. This is the measure's interpretability: “the degree to which one can assign qualitative meaning—that is, clinical or commonly understood connotations—to an instrument's quantitative scores or change in scores” (Mokkink et al., 2010).

The minimal clinically important difference (MCID) is the smallest change in outcome that has clinical value to the stakeholder (e.g., the patient, clinician, caretaker, society) (Wyrwich and Wolinsky, 2000; Eisen et al., 2007). For a measure to be useful for evaluation, its SDC must be smaller than the MCID (Hébert et al., 1997; Beckerman et al., 2001; de Vet et al., 2006). As a field, we have not established a MCID for neurophysiologic outcomes. Establishing an MCID would require the linking of TMS measures to clinical outcomes of interest, which is different from linking them to their mechanistic underpinnings (i.e., validation).

Additional discussion about TMS validity, responsiveness, and interpretability can be found in Supplementary Materials.

## Conclusion

In the present study, we assessed the reliability of common TMS measures obtained from the FDI, with a focus on their potential evaluative and diagnostic applications in healthy older adults and subacute and chronic stroke patients. To determine whether TMS measures could be used to reliably evaluate change, we estimated their SEMeas and SDC. In all subject groups and for all measures, we found that SDCs at the single-subject level were prohibitively high. Thus, these TMS measures cannot be reliably used as a biomarker to assess individual change. However, SDCs become reasonably low with modest sample sizes, justifying use of the measures to detect group-level change. We provide measures' SDC_indiv_ and instructions for calculating SDC_group_, expressly so that future investigators can estimate the SDC needed for pre-post testing in their particular sample. To determine whether TMS measures could be used to reliably discriminate between patients, i.e., to diagnose or stage them, we estimated the measures' reliability_MP_. Although most measures' ICCs were variable across groups, they were universally high for a subset of measures [rMT, TS_MSO_, RC S_50_, RC plateau, and ln(LICI)]; in samples with similar heterogeneity as ours, these TMS measures can reliably discriminate between patients. TMS measures should thus be used based on their reliability in particular contexts. More work establishing their validity, responsiveness, and clinical relevance is still needed. TMS, despite its longstanding use, remains to be fully vetted to have a place at the health outcomes table.

## Author contributions

Author contributions were as follows: study conception and design (HS, JK, DE); data acquisition (HS); data analysis (HS, RO, IM, XL, YC, AR); data interpretation (HS, RO, XL, YC); and drafting/revising the manuscript (HS, RO, DE, JK).

### Conflict of interest statement

The authors declare that the research was conducted in the absence of any commercial or financial relationships that could be construed as a potential conflict of interest.
