# Supplementary material for: The reliability of repeated TMS measures in older adults and in patients with subacute and chronic stroke
Source: Front Cell Neurosci. 2015 Sep 1;9:335. doi: 10.3389/fncel.2015.00335 (PMC4555014; doi:10.3389/fncel.2015.00335)
Supplement: Supplementary file 1 [file SupplementaryMaterials.DOCX]

**The reliability of repeated TMS measures in older adults and in patients with subacute and chronic stroke**

Heidi M. Schambra, R. Todd Ogden, Isis E. Martínez-Hernández, Xuejing Lin, Y. Brenda Chang, Asif Rahman, Dylan J. Edwards, John W. Krakauer

**Supplemental Materials: Further discussion of TMS validity, responsiveness, and interpretability**

***Validity and Responsiveness***

Once reliability has been defined for TMS measures in specific populations, can we freely use the TMS measures to tell us about neurophysiology in these groups? The answer hinges on their validity—how much they actually measure the thing they are purporting to measure (Mokkink, Terwee et al. 2010). Like reliability, validity is a fundamental quality domain of a measurement instrument, and high validity is required to justify its use. An instrument’s reliability is necessary but not sufficient to determine its validity (Portney and Watkins 2009). Though not the focus of this study, meeting the requirements necessary to deem a TMS measure valid is a formidable but critical next step for the field.

In the absence of a gold standard against which to compare TMS measurements, the most pertinent type of validity for TMS is construct validity (Terwee, Bot et al. 2007, de Vet, Terwee et al. 2011). This pertains to whether a measure dependably generates measurements consistent with our understanding of the construct being measured (de Vet, Terwee et al. 2011). In other words, do TMS measurements track with the expected neurophysiology? For example, does a subject with high GABAergic tone always demonstrate stronger SICI? Pharmacological manipulation is a positive first step (Ziemann 2004), but the degree and breadth of neurotransmitter change should be delineated before an absolute relationship to neurophysiology is concluded. Once a measure’s reliability is charted, fluctuations in actual neurophysiology should read out as fluctuations in TMS measurements; sameness in neurophysiology should read out sameness in TMS measurements (Borsboom, Mellenbergh et al. 2004).

The same concepts apply for ascertaining the responsiveness, or longitudinal validity, of the measure. Responsiveness is the validity of the *change*; that is, whether an instrument detects true changes in the construct being measured (Mokkink, Terwee et al. 2010, de Vet, Terwee et al. 2011). Changes in underlying neurophysiology should be reflected as changes in TMS-elicited measurements. Approaches to evaluating construct validity and responsiveness for an instrument are outlined elsewhere (de Vet, Terwee et al. 2011).

It is also important to recognize that validity and responsiveness are also not intrinsic characteristics of the instrument, but must be qualified by the context in which validity is established, and should be generalized only to a similar population or context (de Vet, Terwee et al. 2011). For example, the validity of various TMS measures in healthy subjects may not hold for disease or stroke. Stroke subjects often require substantially larger stimulation intensities to obey conventional paradigms (Talelli, Greenwood et al. 2006), for example the ~1 mV TS_MEP_ required for paired-pulse paradigms. This was evident in the significantly higher TS_MSO_ required in the lesioned hemisphere of our chronic stroke subjects (Table 3). In these individuals stimulated at higher intensities, exactly what physiology are we probing? Validation approaches in patients may help clarify whether we are testing the same neurophysiology that has been altered by stroke, or are testing distinct cortical circuitry with effectively different stimulation parameters.

***Interpretability of measurements in the clinical context***

Once the reliability, validity, and responsiveness of the measurement instrument have been established, and a measurement change exceeds the SDC, one must decide whether the change has any real clinical meaning. This is the measure’s interpretability: “the degree to which one can assign qualitative meaning–that is, clinical or commonly understood connotations—to an instrument’s quantitative scores or change in scores” (Mokkink, Terwee et al. 2010). Interpretability is not a property of the measure, like reliability and validity, but is rather the clinical significance of its measurement (de Vet, Terwee et al. 2011). Critically, if the measurement has no clinical relevance, it should not be used for assessing health outcomes.

The minimal clinically important difference (MCID) is the smallest change in outcome that has clinical value to the stakeholder (e.g., the patient, clinician, caretaker, society) (Wyrwich and Wolinsky 2000, Eisen, Ranganathan et al. 2007). For a measure to be useful for evaluation, its SDC must be smaller than the MCID (Hebert, Spiegelhalter et al. 1997, Beckerman, Roebroeck et al. 2001, de Vet, Terwee et al. 2006). It has been argued that the upper limit of the SDC is the lower limit of the MCID (Hebert, Spiegelhalter et al. 1997); that is, a change must exceed the measurement noise to even begin considering it truly clinically meaningful. As a field, we have not established a MCID for neurophysiologic outcomes. Establishing an MCID would require the linking of TMS measures to clinical outcomes of interest, which is different from linking them to their mechanistic underpinnings (i.e., construct validation).

In the future, TMS measures may be found to be best suited to inform us about a general clinical state but are limited in their mechanistic elucidation (Bestmann and Krakauer 2015). An analogous situation would be a clinical scenario in which a patient has increased 3°C from their normal oral temperature. Although this change exceeds the SDC of the thermometer and the MCID for a temperature change, one can only know that the patient is clinically abnormal. One cannot infer the etiology of the clinical state from the temperature change alone (i.e., infection, malignant hyperthermia, or a recently imbibed hot drink). If infection is the culprit, one cannot estimate pyrogenic cytokine concentration or hypothalamic function from the temperature. Further clinical workup is necessary to identify the source of change, and the quantification of the pathophysiology. Similarly, TMS measures may be found adept at identifying changes in a general clinical state but not in specific neurophysiological circuitry (Bestmann and Krakauer 2015). For example, changes in SICI may reflect an ongoing process, such as the motor cortex engaged in learning (Liepert, Classen et al. 1998, Perez, Lungholt et al. 2004), with the underlying neurophysiological mechanisms driving the learning requiring separate methodological unpacking.

References:

Beckerman, H., M. E. Roebroeck, G. J. Lankhorst, J. G. Becher, P. D. Bezemer and A. L. Verbeek (2001). "Smallest real difference, a link between reproducibility and responsiveness." Qual Life Res **10**(7): 571-578.

Bestmann, S. and J. W. Krakauer (2015). "The uses and interpretations of the motor-evoked potential for understanding behaviour." Exp Brain Res **233**(3): 679-689.

Borsboom, D., G. J. Mellenbergh and J. van Heerden (2004). "The concept of validity." Psychol Rev **111**(4): 1061-1071.

de Vet, H. C., C. B. Terwee, R. W. Ostelo, H. Beckerman, D. L. Knol and L. M. Bouter (2006). "Minimal changes in health status questionnaires: distinction between minimally detectable change and minimally important change." Health Qual Life Outcomes **4**: 54.

de Vet, H. C. W., C. B. Terwee, L. B. Mokkink and D. L. Knol (2011). Measurement in medicine : a practical guide. Cambridge ; New York, Cambridge University Press.

Eisen, S. V., G. Ranganathan, P. Seal and A. Spiro, 3rd (2007). "Measuring clinically meaningful change following mental health treatment." J Behav Health Serv Res **34**(3): 272-289.

Hebert, R., D. J. Spiegelhalter and C. Brayne (1997). "Setting the minimal metrically detectable change on disability rating scales." Arch Phys Med Rehabil **78**(12): 1305-1308.

Liepert, J., J. Classen, L. G. Cohen and M. Hallett (1998). "Task-dependent changes of intracortical inhibition." Exp Brain Res **118**(3): 421-426.

Mokkink, L. B., C. B. Terwee, D. L. Patrick, J. Alonso, P. W. Stratford, D. L. Knol, L. M. Bouter and H. C. de Vet (2010). "The COSMIN study reached international consensus on taxonomy, terminology, and definitions of measurement properties for health-related patient-reported outcomes." J Clin Epidemiol **63**(7): 737-745.

Perez, M. A., B. K. Lungholt, K. Nyborg and J. B. Nielsen (2004). "Motor skill training induces changes in the excitability of the leg cortical area in healthy humans." Exp Brain Res **159**(2): 197-205.

Portney, L. G. and M. P. Watkins (2009). Foundations of clinical research : applications to practice. Upper Saddle River, N.J., Pearson/Prentice Hall.

Talelli, P., R. J. Greenwood and J. C. Rothwell (2006). "Arm function after stroke: neurophysiological correlates and recovery mechanisms assessed by transcranial magnetic stimulation." Clin Neurophysiol **117**(8): 1641-1659.

Terwee, C. B., S. D. Bot, M. R. de Boer, D. A. van der Windt, D. L. Knol, J. Dekker, L. M. Bouter and H. C. de Vet (2007). "Quality criteria were proposed for measurement properties of health status questionnaires." J Clin Epidemiol **60**(1): 34-42.

Wyrwich, K. W. and F. D. Wolinsky (2000). "Identifying meaningful intra-individual change standards for health-related quality of life measures." J Eval Clin Pract **6**(1): 39-49.

Ziemann, U. (2004). "TMS and drugs." Clin Neurophysiol **115**(8): 1717-1729.
